# PPARs/RXRs in Cardiovascular Physiology and Disease

**DOI:** 10.1155/2008/173780

**Published:** 2008-03-31

**Authors:** Brian N. Finck, Giulia Chinetti, Bart Staels

**Affiliations:** ^1^Department of Medicine, School of Medicine, Washington University, 660 South Euclid Avenue, Campus Box 8031, Saint Louis, MO 63110, USA; ^2^INSERM U545, Institut Pasteur de Lille, Faculté de Pharmacie et Faculté de Médecine, Université de Lille 2, 59000 Lille, France

The PPAR family of nuclear receptor transcription factors are important regulators of cardiovascular function and metabolism. Because of this, PPARs are potentially interesting pharmacologic targets for treating cardiometabolic disease. The reviews in this series discuss the regulatory functions of PPARs in maintaining metabolic and physiologic homeostasis in a variety of cells and tissues. Additionally, the therapeutic potential and mechanisms of action of ligands of the different PPAR isotypes are discussed.

The review series is started by an examination of the effects of PPARs on lipoprotein metabolism. This is one of the first identified functions of PPARs. Indeed, ligands for PPAR*α* were in clinical use as lipid-lowering agents even before their pharmacological target, PPAR*α*, were known. The second review evaluates the important anti-inflammatory effects of PPARs in platelets, which is emerging as an important mechanism of their beneficial effects. Next, the critical role that PPAR*α* and its transcriptional coactivator protein PGC-1*α* play in regulating energy metabolism and function of the myocardium is discussed. Then, a series of reviews focuses on the potentially beneficial effects of PPAR*γ* agonists on the cardiovascular system. Several aspects are presented. The effects of PPAR activation on the cardiovascular system as a whole, on the vascular smooth muscle cell, and in the context of diabetic cardiovascular disease are each discussed at length. A review by Demers et al. also discusses the potential input of the hexarelin signaling pathway in regulating PPAR*γ* activity and its potential impact on cardiometabolic disease. The genes encoding the PPARs are rich with genetic variation and the impact of these polymorphisms and haplotypes on the response to PPAR activators is only beginning to be understood. Thus, the “pharmacogenomics” of PPARs are discussed in a review by Dr. Sharon Cresci. Finally, the potential toxicity and adverse outcomes of PPAR agonism are summarized in detail by Jennifer Robinson. The timeliness of this discussion is outstanding given the recent reports of increased cardiovascular morbidity associated with use of rosiglitazone and the failure of PPAR dual agonists at different stages of development. Several of the other reviews in this series also touch this controversial issue at least briefly.

We are also pleased to present two original research reports. The first report found associations between PPAR*γ* gene polymorphisms and several cardiometabolic indices, but found no link with cardiovascular morbidity and mortality. Second, Buroker et al. report an important role for PGC- 1*α* in postnatal metabolic maturation. This preprogrammed burst in cardiac oxidative metabolism is an important developmental response that also has implications for other physiologic states wherein the demand for ATP production is rapidly induced.

We hope that you will find this issue enjoyable and informative.

*Brian N. Finck* *Brian N. Finck*

*Giulia Chinetti* *Giulia Chinetti*

*Bart Staels* *Bart Staels*

